# On the Use of Water in Surgery

**Published:** 1851-06

**Authors:** Alphonse Auguste Amussat

**Affiliations:** Paris


### BUFFALO MEDICAL JOURNAL

AND

### MONTHLY REVIEW.

**VOL. 7.** JUNE, 1851. **NO. 1**

### ORIGINAL COMMUNICATIONS.

ON THE USE OF WATER IN SURGERY. *By Alphonse Auguste Amussat, of Paris ; published at Paris, Jan,* 1851, *and translated from the French, by Frank H. Hamilton, Prof. of Surgery in the University of Buffalo.*

(*Continued from page* 726.)

### CHAP. II.

#### GENERAL CONSIDERATIONS UPON THE USE OF WATER.

In the first chapter of this work, I have collated all the historical documents which I could procure, on the use of water in surgery.

In this second chapter I shall enter upon the practical part of my subject, and I shall examine the following questions:

1. What kind of water ought to be used in surgery?
2. What are the effects of water at different temperatures?
3. What, in general, is the most suitable temperature?
4. How long ought the water to be continued?
5. What are the advantages of the use of water at a suitable temperature?
6. What are the inconveniences which have been attributed to this agent?

After this general examination, I will describe in the following chapter the principal modes which have been indicated for the application of water.

1. *What kind of water ought to be used in surgery.*

We may employ every kind of *fresh* water, and consequently that which is most easily to be procured; but if we have our choice, we should prefer such water as contains the smallest quantity of saline and foreign particles.

Earthy and saline waters, when long continued, deposite upon the epidermis much salts, which form a kind of adherent crust, as we have lately noticed in the case of a lady whom we submitted, for more than two months, to continued irrigations with tepid well-water, for a fracture of the leg with a large wound and gangrene of the integuments.

2. *What are the effects of water at different temperatures?*

Cold water, that is, from 10° down to 0 C, (50° to 32° F,) or to the freezing point, applied continuously upon inflamed parts, is a powerful antiphlogistic and sedative, when the patients can support it without inconvenience. It abates the morbid local heat, and subdues the inflammation, one of whose chief elements is now removed; it determines in the tissues, at the point of its application a fibrillary contractility, which constringes the structures, and augments, so to speak, their cohesion; it possesses, then, a very decided astringent property. By diminishing the calibre of the capillaries it repels the blood and becomes repercussive. These properties have often been put to profit in arresting hemorrhages, and in diminishing the too great laxity of the skin in certain parts, as in the superior palpebræ, scrotum, &c.

Cold water has also the property of directly exciting the tissues, and with an energy proportioned to the lowness of the temperature, and the suddenness of the impression. We may invoke this property of cold water advantageously, in all cases of atony, and of debility of any part of the system, to arouse action and to restore the tissues to the condition of excitement necessary to cicatrization.

M. Pirogoff affirms that "there is no better means than the employment of ice or snow, to combat the scorbutic tendency which a low temperature impresses upon wounds during the rigorous winters, so common in Russia." "I do not know," says M. Richet, from whom I borrowed this passage, "whether the Russian surgeon applies the cold intermittently; yet in this way alone can I explain its efficacy, by supposing its success to depend upon the reaction which always results from its sudden application and removal."

I think, with M. Richet, that it is by the momentary application of very cold water upon the surface of the tissues, securing thus its stimulating properties, that M. Pirogoff obtains his success in all the cases above alluded to.

The surgeons who have used cold water most, have constantly noticed a depression of the temperature of the skin; but no precise experiments have been made to ascertain whether there was an equilibrium of temperature between the liquid and the skin.

What has been said of cold water, is yet more manifestly true of ice and of freezing mixtures.

"The mode of action of cold water," says Lombard, "being known by daily experience upon sound parts, it is easy to understand what must be its effects upon wounds in general; and it is proper to say, that during the access of the inflammation, to which contused and lacerated wounds are principally subject, it is absolutely necessary to repeat the application often, to prevent *the increase of heat and the desiccation.* The coolness of the water also, while it tempers the heat, obviates congestions in the affected vessels. Consequently the suppuration is infinitely less and more prompt as the following history will prove.

"Christophe Hebert, a fusileer in the regiment of Alsace, company of Ruttenburg, aged about twenty years, of a delicate temperament, received the 9th of February, 1785, a cut from a knife which severed the extensor tendons transversely, and also the metacarpal bones which sustained the three last fingers. He was immediately taken to the hospital.

"The mode of dressing consisted in placing the hand upon a pallet, so that, by the aid of a crucial bandage, we could retain the fingers in place after they had been adjusted. This appareil was simply moistened with cold water, with express injunctions to renew it as often as the patient discovered in the limb a certain degree of heat. The hæmorrhage, although quite copious at first, soon ceased. On the third day, the hand was a little swollen, but the pain was so slight as not in the least to discompose him, or disturb his sleep, and the wound had such a healthy secretion, as to permit us to regard the swelling as of no consequence. Suppuration progressed regularly without becoming excessive, and was at all times healthy. The dry lint, employed in the last dressings, completed the cicatrization which the continued use of cold water had so well commenced, and the soldier left the hospital on the 19th of March, perfectly cured, and without having experienced a single unfavorable symptom."

Cold water possesses very marked stimulating properties; it congests and reddens healthy tissues, and it produces the same phenomena upon inflamed parts. It also hastens suppuration.

At the temperature of 30° to 35° C, (86° to 95°F,) that is to say, below and near that of the body, its application upon an inflamed part, where pain, heat, swelling, and redness are present, relaxes the tissues, obtunds their excessive sensibility and contractility, and in a word, produces marked relief. It the application is continued, the cortege of inflammatory symptoms are seen little by little to disappear, and its emollient action is exhibited in the most favorable manner. At this temperature, also, it promotes suppuration. We see indeed every day, wounds whose appearance was satisfactory, and which had reached the stage of regular suppuration, suddenly assume, under the operation of various causes, a bad aspect, in connection ordinarily with a manifest diminution, and sometimes with an arrest of the secretion of pus. In these cases the employment of water moderately warm soon restores the secreting surfaces to their normal conditions.

Water, at a middle temperature, that is to say between 18° and 25° C, (64° and 77° F,) partakes of the properties of cold water and of warm. At this temperature it has almost all the advantages of cold water without its inconveniences, as we shall hereafter consider.

Its use, after an operation, destroys the erethism and the pain which ordinarily succeeds; if on the contrary, the patient is in that state of stupor which usually accompanies grave lesions, such as violent falls, crushing by machines, wounds from fire-arm, &c., it restores tranquillity by regulating the action of the nervous system.

In cases where inflammation has not yet occurred, it prevents it, and often obviates the development of traumatic fever. If, however, these phenomena are already manifest, water moderates them, or makes them entirely cease, and arrests the accidents which the inflammation has produced. In short, its local effect seems to be to diminish the tendency of innervation to determine to one point all the elements of inflammation.

I have witnessed the power of continued irrigations of tepid water, in subduing traumatic fever, consequent upon a very severe operation made by my father upon a lady sixty years old. Water at 20° or 22° C, (68° or 71° F,) employed immediately after the operation had prevented all inflammatory reaction, when the patient suspended the application during the night. Fever was soon apparent. After several hours the irrigations were recommenced, and the fever ceased. Again the water was suspended and the fever reappeared, and again applied and it disappeared.

In the treatment of simple wounds, water favors greatly union by first intention, as has been already noticed by A. Berard.

If we carefully note the action of water upon a suppurating surface, we see that it acts as an antiphlogistic and detersive. It moderates the purulent secretion and removes the pus as fast as it is produced. Indeed we may affirm, that from the use of this agent there results a suppuration laudable in quality and in quantity; also an inodular membrane, and by consequence, a cicatrix, formed with more regularity and promptness.

How does water produce the effects of which we have spoken?

Is it by restraining the morbid development of caloric in the part? (Martel, Parr, Josse.)

Is it by a hyposthenic action upon the nervous radicals?

Is it by absorption, as Louis, Percy and Lombard think, in opposition to M. M. Josse and Malgaigne?

Is it because water by irrigation acts upon the parts as a bath of moderate temperature, which, while it does not interrupt the circulation of the fluids, prevents nevertheless their being carried in too great abundance to the diseased limb? (Nivet.)

Is it by maintaining in the diseased parts a uniformity of temperature, that the cataplasms and warm or hot water dressings of Mr. Liston act, producing an incubation of a novel kind? (Richet.)

Is there a developement of electric phenomena, from the contact of the water with the body at unequal temperatures? (Josse.)

The subtraction of morbid caloric, and the depression of the temperature upon the surface where the water is in contact with our bodies, are phenomena which we understand. But as to the other questions, they are yet in a state of hypothesis; nevertheless, I am inclined to think that they also have some connection with the truly marvelous effects produced by water.

Relative to absorption, the following experiments, made by M. Patry, incline us to admit it as an explanation, at least under some circumstances. "To determine," says he, "the *modus operandi* of cold water there is an experiment which I have many times repeated : If we apply a vesicatory, and remove it when rubefaction is produced, water, upon first contact with the inflamed skin, determines a painful and smarting sensation; but soon the pain is blunted, the sensation of burning heat diminishes and at length ceases entirely. If we suddenly suspend the application of the liquid, the inflammation is promptly renewed, and with more violence than at first; for the vascular ramifications having become more permeable admit now a larger quantity of blood. Again, if we remove the vesicatory when the vesicles have begun to form, we see them diminish and even completely disappear some hours after the application of cold water. If we suddenly cease the application, the vesicles are soon seen to be reproduced and to augment in volume. This fact demonstrates that the physiological action of absorption is not retarded by cold water applied to the surface of wounds.

"To the patient who was the subject of the first observation, I have several times applied three centigrammes of sulphate of morphia upon the surface of the wound during continued irrigation; soon he has become drowsy. The following day I have substituted ten centigrammes of tartrate of antimony for the morphine, and the patient has experienced nausea without vomiting.

"A woman complained of a very severe rheumatic pain, seated in the deltoid muscle. Several blisters had been in vain applied upon the painful spot. The epidermis was removed after the last blister, and the raw surface sprinkled with three centigrammes of morphine, and then covered over with wet lint; the lint was constantly moistened. Night and morning the morphine was renewed, and the fourth day the pain had completely gone.

"These facts prove, beyond controversy, that the application of cold water to the surface of a wound, does not hinder absorption. We should, nevertheless, take care when desiring the absorption of a remedy, not to pour upon the part an excess of water, for, whether soluble or not, it will be washed off.

"If we remove the vesicatory when the cuticle is already very much raised, absorption will not take place, however long the water may be applied.

"Upon all the patients, whose cases I have cited in this memoir, I have established the action of absorption by the application of cold water to the surface of the wound."

Malgaigne, after having recalled the different theories which have been devised to explain the action of irrigations, says:

"Without engaging in a discussion which is scarcely susceptible of an entirely satisfactory termination, I will say that in my opinion, the only way of comprehending the therapeutic action of an antiphlogistic, is to recognize in inflammation several elements, according to the doctrine of Berard and Barthez. If we attack one of these elements before the inflammation is developed, frequently it is entirely prevented; if after it is established, the success will depend upon the relative value of the element attacked, and of the stage at which the inflammation has arrived. Thus opium in large doses, by subduing the pain, obviates phlogosis; while leeches act by diverting the sanguinary afflux.

"So by cold irrigations, in whatever way applied. we combat directly, either before or after their appearance, the heat, the sanguinary afflux, &c. With certain subjects, however, other elements predominate, as for example, the nervous element or pain, then cold irrigations are powerless, *and it becomes necessary to resort to tepid liquids.*"

3. *What, in general, is the most suitable temperature?*

This question, which addresses itself to every surgeon who resorts to the therapeutic agent of which we speak, is far from being definitely answered; it even appears to me that the ancients had a better appreciation of the value of temperature than the moderns, for they recommended that the water should be warm in winter and cold in summer, yet this generally had reference to affusions or fomentations, whose action is less powerful than irrigations and immersions.

"Cold is severe upon wounds," says Hippocrates; "it hardens the skin, excites pain, retards suppuration, predisposes to gangrene, shiverings, convulsions, tetanus."

The ancient school, imbued with these principles, showed itself in general more favorable to the employment of water at a mild temperature, and even warm rather than cold.

If we refer to the historical part of this work, we shall observe that A. Pare, Lamorier, Louis, Pontier, Guyot, Recollin, and Chambou generally gave the preference to tepid water.

Lombard, who has studied with care the differential action of water in the treatment of a great number of surgical affections, thus expresses himself: "The varieties of which diseases are susceptible, according to the person affected; the different conditions into which they pass successively, depending upon the age, the time, the season and the place; the parts more or less delicate which are involved, etc., are circumstances which do not permit the supposition that cold water can be a remedy applicable to every kind of injury.

" Such are the general properties of tepid water, that it softens the tissue of the skin, relaxes the texture of the nerves which are distributed through it, and dilates the pores and prepares them to receive the smaller particles of water, to be afterwards carried along in the course of the circulation. These various effects, contrasted with those invariably produced by cold water or ice, teach us the necessity of managing these topical applications according to the relative indications.

" In no way can we combat inflammation more effectually, in no way can we relieve the maladies of persons of a dried-up constitution, and especially of old men, whose fibres are stiff and withered, than by baths, fomentations, immersions, and ablutions of tepid water. The true and only remedy is, to preserve the flexibility and the action of the fibres, by moistening them as often as possible.

"The most skillful practitioners of the last centuries did not confound the properties of cold water with those of warm. The proper time for employing this remedy, cold or warm, was indicated by the changes which occurred in the wounds, and by the different revolutions which each season brought with it. The modifications of heat and of cold, of which water is susceptible, seemed to fulfill all indications. *The degree of heat at which they ordinarily employed water, was such as would excite an agreeable sensation when the hand was plunged into it.* This is what Pare intends when he says that it ought to be temperate or tepid."

In support of these ideas, the author cites the following facts:

"Two days," says he, "after the entrance of the soldier from Alsace into the hospital, there presented himself a corporal of the regiment of Foix, company of La Richardiere, named Guirdin, wounded by a thrust from the point of a sabre, which traversed the thickest part of the deltoid. This man was of a bilious complexion, and had a very irritable fibre. The excessive tumefaction which occurred in the shoulder, the moment the wound was received, indicated an effusion in the track through which the sabre had passed. To evacuate the blood, I enlarged the posterier opening, whose narrowness did not permit it to escape. This done, I had the tumor fomented with *warm water*, and soon it was diminished more than half, the blood having escaped freely from under the gentle warmth maintained by the fomentations. The track closed gradually, with slight suppuration, and the two wounds cicatrized promptly. The progress of cure was steady and uninterrupted, and the patient resumed his military exercises on the twenty-fourth of March.

"The twentieth of April following, two fusileers of the regiment of Hesse Darmstadt, entered the hospital, wounded by blows inflicted for desertion. One of them, twenty-two years old, was of a humid temperament, and had a very loose fibre; the other, in his thirty-eighth year, was, on the contrary, of a robust constitution, bilious and dry.

"The first, Jean H———, was constantly dressed with *cold water*, and was cured on the ninth day, although the integuments had been lacerated very deeply in several places.

"The second, Christophe A———, was fomented with *warm water:* and notwithstanding he had been more injured than the first, he was entirely cured on the tenth day."

Lombard has, as we see, endeavored to ascertain the difference in the effects of cold and tepid water.

I partake of his opinion and of that of A. Pare, as to the estimate we should place upon tepid water, and to this I have been led by observing the cases occurring in the practice of my father, which, while they demonstrate how easily cures are effected with tepid water, which is exempt from the inconveniences charged to cold, furnish proofs at the same time that tepid water should be generally employed.

Percy, Briot, Guthrie, &c., employed according to circumstances, either cold or tepid water: but as they used it generally upon the field of battle, with great numbers of wounded to dress, and destitute often of the most necessary conveniences, they resorted more frequently to cold water, because it was most easily obtained.

"Tepid water," says Sanson, "possesses especially emollient properties in an eminent degree: applied to the healthy tissues, it is promptly absorbed by them, swelling and softening them, without attracting the blood, so that they are left pale and colorless; applied to inflamed tissues, it relaxes them, and *perhaps by facilitating the circulation of the accumulated blood*, it assuages the pain and hastens resolution. It acts as a cataplasm, or rather, as the advocates of water say, cataplasms act as curative agents only by virtue of the water which they contain, while simple water possesses the advantage over cataplasms of being more readily absorbed, since it does not form upon the skin a mucilaginous layer which obstructs absorption; it undergoes no change: it is less cumbersome, less expensive, it is found everywhere, and is subject to this one inconvenience alone which does not belong to cataplasms, namely, it must be more frequently renewed."

Most surgeons who have advocated water as a means to combat inflammation, have generally used it cold, partly because they considered morbid heat as the element especially dominant in inflammation, and partly because it was more easily obtained ; and it is but just to say, that success has attended, and will often continue to attend, this mode of treatment.

Nevertheless, when we analyze the ill effects which have been charged to the use of water in surgery, we can not fail to be convinced that they apply properly not against the liquid itself, but against the too low temperature at which it has been habitually used.

If then we can abstract morbid heat, and subdue inflammation with water at a temperature agreeable to the patient, that is, near yet generally below the temperature of the body, as well as with cold water, we are, I hope, prepared to admit that cold water ought to be employed only as the exception.

However, it will be easily understood that the use of water at this medium temperature, which varies generally from 18° to 25° C. (64° to 77° F.) depends upon the constitution of the patient, the condition in which he is found, the season, the climate, &c. Water at 15° C. (59° F.) appears warm in summer to a sanguine, robust man, suffering under a violent fever, while it will be cold to a nervous debilitated female, or to an infant. We see then at once, that in establishing as a general rule for temperature, the sensations of the patient, we have a thermometer preferable to every other; we can now vary the temperature according to a multitude of particular indications, and thus avail ourselves of all the advantages of water without any of its inconveniences. Such has been the rule of practice adopted by my father and myself, and to the present time we have had no reason to be dissatisfied with it.

I have dwelt the longer upon the question of temperature, because I think it essential to the future reputation of water in surgical therapeutics.

4th. *How long ought the water to be continued?*

The duration of the local application of water will vary not only according to the mode in which it is applied, but also according to the judgment of different surgeons.

Water having been generally employed cold, and under the appellation of an antiphlogistic, its use has been suspended when the inflammation was subdued, and there existed no farther probability of its return.

At the hospital of the University of London, water, which is considered as an antiphlogistic, and also as the best topical application in most of the sequences of inflammation, is employed generally during all the treatment.

Since the success of Priessnitz, cold water constitutes the burden of the whole treatment of all the patients in hydropathic establishments.

I think, with my father, that water, employed in an appropriate manner and according to the different modes which I shall hereafter describe, is alone sufficient for a great number of surgical maladies from the beginning to the cure.

5th. *What are the advantages of the use of water at a suitable temperature?*

We may regard water as the most powerful of all local antiphlogistics, and by proper variations in temperature and in modes of application, it may be rendered sedative, emollient, excitent, astringent, repercussive, &c.

It offers the farther advantage of diffusing and carrying off secretions which excoriate the tissues, or which, by infiltrating into their structure, often caused disorganization and death.

By maintaining in the wounds an inflammation which does not exceed proper limits, and by removing the pus as fast as it is formed, it favors union by first intention in simple wounds, and in many cases it obviates that excessive secretion of pus which exhausts the patients; it also prevents purulent absorption, hospital gangrene, &c.

Properly applied, it maintains the parts at a uniform temperature, which circumstance goes far toward the accomplishment of a cure.

"One of the principal advantages of simple water over other dressings in common use, such as compound fomentations, cataplasms, and unguents, is its cleanliness, which contributes not a little to the cure. For to render the use of water still more efficacious, it is proper, each time the dressings are renewed, to wipe gently the part with a piece of soft and dry lint. This is considered a point of great importance among skillful surgeons." (Lombard.)

Employed upon a grand scale, in the wards of hospitals, for example, it would prevent the development of those mephitic gases produced by the fermentation of pus, and whose influence is pernicious to the sick.

To all these considerations I will add one more, which is a natural sequence to the superiority of water as a therapeutic agent, to wit, we may nourish our patients at a much earlier period than usual. Finally, I hasten to say, that modern surgery has abandoned the practice of *absolute diet*, which, by enfeebling the system beyond measure, places it in the most favorable condition for purulent absorption, as has been well demonstrated by Majendie.

If I have been able skillfully to present my subject in all its bearings, it will have been seen, I trust, how much preferable, in every class of diseases, and in every point of view, are the improved water-dressings to those now generally in use, and the large number of facts, which can not be published in this memoir, will hereafter be found to sustain this proposition.

I must, nevertheless, be permitted to introduce the opinion of M. Sedillot upon dressings after amputations, because it accords entirely with that to which my own careful investigations have brought me, as to the necessity of simplifying dressings.

In a valuable article on the "*means of insuring success after amputation of limbs*," M. Sedillot thus speaks:

"The dressings, in consequence of the grave accidents to which they give rise, are one of the great causes of the mortality of amputations. The stump is strangulated by unyielding rollers, and the wound by straps and sutures.

"The fluids, blood, serum and pus, imprisoned within the wound, compress the flesh, prevent free circulation, induce œdema, swelling, inflammation, erysipelas, purulent deposits, ulceration, phlebitis, erosions of veins, *pyoemie*, caries and necrosis of the bones, etc. Let every surgeon recall his own experience, and answer, whether at the removal of the first dressing he has not seen the skin œdematous, covered with phlyctenulae in the intervals between the adhesive straps, attacked with an erysipelatous redness, a sanious and fetid pus flowing from the interior of the stump, and all the patients expressing marked relief after the dressing. Who has not been a witness of wounds united almost through their whole extent, in which it became necessary to open them to allow pus, collected in narrow fistulas and large depots, to escape? What abscesses and purulent channels have retarded the healing! What caries and necrosis have indefinitely deferred the cure! Such too frequently are the facts, as may be easily verified in all hospital services: and it is no longer a matter of surprise that physicians are disposed to renew their dressings often in order to save their patients from such severe accidents. I certainly think, if we are to employ dressings at all, it is better to remove them at about twenty-four hours, and assure ourselves of the condition of the stump, rather than wait four or five days in complete ignorance of the state of the wound; but it must be remembered that the renewal of dressing is in itself fatiguing, painful, exposes to cold, and consequently to tetanus; it requires much time, and must be confided to assistants whose experience does not equal their zeal. While they remain upon the wound, a hemorrhage can not be immediately discovered. The amputated limb is either too much or too little compressed, the bandages are relaxed, the flesh is not sufficiently supported, the muscles retract, and in spite of the perfection of the operative maneuver, the bone protrudes, dries, and the life of the patient is endangered.

"A bandage, however well applied, is a feeble measure against the evils of which we have spoken, and the remedy must be more energetic and more complete. If we admit that dressings, whether removed often or seldom, aggravate the dangers, the question is settled: they ought at once to be abandoned. How to dispense with dressings may appear incomprehensible to physicians educated to respect the pledget and the bandage, yet this is a reform which we have adopted, and to which we ascribe our success. But how shall we prevent retraction of the muscles, conicity of the stump, and obtain a cicatrization of the wound? By a means both simple and of easy execution. Dressings are only intended to maintain mechanically in contact, the edges of the wound. If the edges will remain spontaneously in contact, then the dressings are useless, and this is the end which we propose to attain by abandoning the circular amputation, and resorting to a method of one anterior flap, comprising two thirds of the circumference of the limb. The last third is cut perpendicularly from the angle of the flap; the bone is exposed more or less, according to the indications, and the flap falling back naturally upon the wound, by its own weight covers and closes it, without the absolute necessity of any dressing whatever.

"A linen cloth folded double, and of about two fingers breadth, dipped in some digestive, is applied to the end of the bone in such a way as to con_ stitute a central canal for the escape of the liquids. Two pins stick and hold in apposition the angles of the flap, until inflammatory induration has commenced, so as to secure union by first intention upon each side, without the retention of pus, for the removal of the piece of lint from the center, about the third or fourth day, leaves a vertical cavity in which blood, serum, and pus can not accumulate. The stump remains naked, exposed to the eye of the surgeon; and the slightest accidents are at once perceived and submitted to proper treatment; if we wish to apply cold or heat, the wound is always accessable, and may be at pleasure covered with ice or cotton. Fomentations made with square pieces of carded wool, lotions, embrocations, frictions, injections, &c., can be easily employed. The pus diffused upon the diaper cloths, (*drap d' alize,*) does not become offensive, and in case the limb is affected with spasms, it can be confined with a handkerchief, or any other piece of cloth, the ends of which shall be fixed to the bed or to the side of the hoop destined to support the weight of the coverings. We take the precaution, also, to remove the anterior sharp edge of the bone at the place of amputation, in order to prevent the soft parts from being irritated thereby, and the interposition of a piece of linen during the first days, appears to us to contribute to this result. The projection of the bone becomes then impossible, except by penetrating through the whole thickness of the flap, which cannot happen when we take care to cut the bone sufficiently high."

I will add, (and I must here express my regret that I am not able to adopt the particular opinions of M. Sedillot, as to the best mode of dressings after amputations,) that with water dressings of whatever kind, the stump is always in a condition of excessive cleanliness, that the pus is carried off as fast as secreted, and no putrid fermentations can occur. In short, we can, at any moment, examine the wound, without causing the least pain to the patient. The stump, instead of being heated and compressed by compresses and bandages, lies free in a humid atmosphere, whose temperature the surgeon regulates at pleasure.

